# A Review on the Methodology and Use of the Pregnant Mouse Model in the Study of *Brucella* Reproductive Pathogenesis and Its Abortifacient Effect

**DOI:** 10.3390/microorganisms12050866

**Published:** 2024-04-26

**Authors:** Aitor Elizalde-Bielsa, Pilar M. Muñoz, Amaia Zúñiga-Ripa, Raquel Conde-Álvarez

**Affiliations:** 1 Department of Microbiology and Parasitology, Instituto de Investigación Sanitaria de Navarra (IdiSNA), University of Navarra, 31008 Pamplona, Spain; aelizalde.7@alumni.unav.es; 2 Department of Animal Science, Centro de Investigación y Tecnología Agroalimentaria de Aragón (CITA), 50059 Zaragoza, Spain; pmmunnoz@cita-aragon.es; 3 Instituto Agroalimentario de Aragón—IA2, CITA-Universidad de Zaragoza, 50009 Zaragoza, Spain

**Keywords:** *Brucella*, pregnant mouse, model, placenta, trophoblast, reproductive pathogenesis, abortion, vaccine

## Abstract

Brucellosis is one of the most common and widespread bacterial zoonoses and is caused by Gram-negative bacteria belonging to the genus *Brucella*. These organisms are able to infect and replicate within the placenta, resulting in abortion, one of the main clinical signs of brucellosis. Although the mouse model is widely used to study *Brucella* virulence and, more recently, to evaluate the protection of new vaccines, there is no clear consensus on the experimental conditions (e.g., mouse strains, doses, routes of inoculation, infection/pregnancy time) and the natural host reproducibility of the pregnant mouse model for reproductive brucellosis. This lack of consensus calls for a review that integrates the major findings regarding the effect of *Brucella* wild-type and vaccine strains infections on mouse pregnancy. We found sufficient evidence on the utility of the pregnant mouse model to study *Brucella*-induced placentitis and abortion and propose suitable experimental conditions (dose, time of infection) and pregnancy outcome readouts for *B. abortus* and *B. melitensis* studies. Finally, we discuss the utility and limitations of the pregnant mouse as a predictive model for the abortifacient effect of live *Brucella* vaccines.

## 1. Brucellosis and *Brucella* Reproductive Pathogenesis

Brucellosis is one of the most common and widespread bacterial zoonoses and is caused by bacteria belonging to the genus *Brucella*. This Gram-negative bacterial genus belongs to the Class α-2 of the Phylum *Proteobacteria* and includes a variety of species that exhibit a wide diversity of preferential hosts and zoonotic potentials. Classical brucellae, the most epidemiologically relevant *Brucella* species, infect domestic ruminants (*B. melitensis*, sheep and goats; *B. abortus*, cattle; and *B. ovis*, sheep), swine (*B. suis* biovars 1-3), and dogs (*B. canis*) [1]. Nonetheless, the already mentioned host preference of the different *Brucella* spp. does not mean host restriction: *B. melitensis* can also infect cattle and other ruminants; *B. abortus* infects buffaloes, camels, deer, dogs, horses, goats, and sheep, whereas *B. suis* infects cattle, horses, rabbits, and dogs [2]. These species are the main causative agents of human brucellosis (except for the non- or low-zoonotic *B. ovis*, *B. suis* biovar 2, and *B. canis*); *B. melitensis* is the most important species worldwide.

The pathology in the natural host features a wide range of reproductive symptoms [2,3]. Infection may result in infertility, reproductive failure with abortions/stillbirths, or the birth of weak offspring, all related to the development of placentitis [4,5,6] and reduced milk production due to infection and inflammation of the mammary glands [5,7,8]. 

In this context, human infection is acquired through contact with infected animals and the consumption of unpasteurised milk products. Therefore, brucellosis is an occupational risk for veterinarians, slaughterhouse workers, butchers, and livestock farmers and a public health threat in populations consuming raw milk or unpasteurised dairy products [9]. Although under-reporting is common and official records are of questionable value in resource-limited countries, the available evidence suggests that brucellosis is emerging or re-emerging in many regions as a result of both the increased demand for animal products, leading to intensification of livestock production, and poor understanding of the disease and control measures [10,11,12,13,14,15,16,17]. A recent worldwide study has provided an empirical estimate of between 1.6 and 2.1 million new cases of human brucellosis annually, most of them in Africa and Asia, but also occurring areas within the Americas and Europe [18]. The economic impact of brucellosis depends on the prevalence, species affected, management, sociopolitical decisions, trade restrictions, and the extent of human disease and is considered to be very important in low-income countries around the world [10,15,19,20]. Human-to-human transmission is negligible, and control of the disease relies on animal vaccination. Currently, the only effective vaccines are live attenuated strains, which have several drawbacks such as an abortifacient effect when administered to pregnant animals, similar to that of wild-type strains [21], imposing a great barrier to the application of mass vaccination campaigns. For this reason, there is a clear need for research into the development of reproductively safe *Brucella* vaccines. 

**Figure 1:**
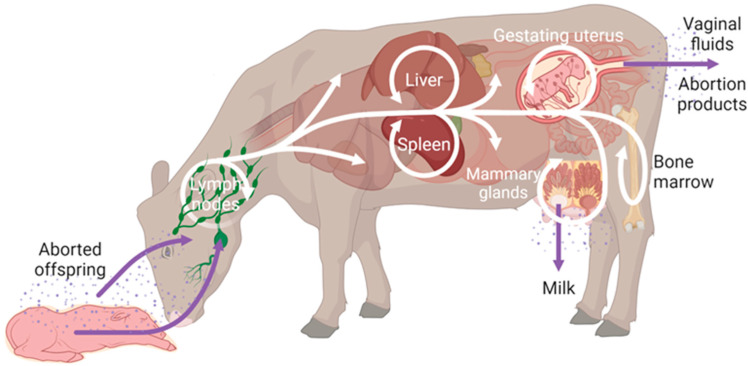
Schematic representation of the main ports of entry, dissemination and persistence foci, and excretion of *Brucella*. The main way of transmission for brucellae is through aerosols, for example through contact with abortion products with massive amounts of bacteria per gram. Hence, brucellae access the organism mainly through the oral and nasal mucosae, where they are internalised by phagocytes that fail to clear the infection. *Brucella* survive and replicate within these cells and, as a consequence, they disseminate through the organism, developing into a systemic infection. This way, *Brucella* can access their preferential organs for replication, such as the spleen or the reproductive organs (e.g., the placenta), resulting in abortion and excretion in the milk in females, further transmitting the infection to new individuals. (Created with Biorender.com).

Transmission in animals usually occurs through the head mucosae by contact with contaminated placentae, genital secretions, or aborted foetuses or ingestion of contaminated milk [5,7,22,23]. Thus, the principal port of entry for *Brucella* are the mucosal barriers, mainly the nasal and oral mucosae [24] (Figure 1). Here, brucellae are internalised by cells of the mononuclear phagocyte system, where they avoid intracellular killing and multiply in high numbers in a compartment derived from the endoplasmic reticulum (ER) [25,26,27,28]. From the lymph nodes, brucellae disseminate systematically to other lymphoid tissues, such as the spleen [29], and to reproductive organs such as the gravid uterus and the placenta [30] (Figure 1).

As mentioned above, brucellae have a predilection for the placenta in ruminants, leading to placentitis, which impairs nutrient delivery to the foetus and induces foetal stress, a phenomenon that has been recognised for decades [31]. This, along with foetal infection, has been hypothesised as the cause of abortions and perinatal deaths [31,32,33,34]. In pregnant ruminants, around 85% of brucellae are found at high numbers in placental structures, especially at the cotyledons, with approximately 10^13^ CFU/g in bovine or 10^8^ CFU/g in caprine species [34,35]. Such high numbers of brucellae in reproductive and aborted tissues ensure transmission via aerosols, ingestion, or sexual intercourse [30]. Therefore, this abundant multiplication in the reproductive tract of the natural hosts is crucial in the biology of *Brucella*, a very efficient strategy for bacteria to spread to new hosts [31].

Placentitis is considered a consequence of the inflammation and necrosis caused by the death of infected placental trophoblasts, in which *Brucella* are found intracellularly [34,36,37,38,39,40,41]. This trophoblast necrosis in infected placentae is different from the behaviour of *Brucella* in the mononuclear phagocyte system, whose cells appear apparently unaware of the presence of the parasite [31,42,43,44,45]. In fact, classical *Brucella* strains inhibit the death of these phagocytic cells [43,46].

In a *B. abortus* intravenously (IV)-infected pregnant goat model, the access to the placenta was shown to occur at the level of the erythrophagocytic trophoblasts [34], with subsequent spreading to the cells of the chorioallantoic membrane, preferentially trophoblasts. Similarly, Payne reported that brucellae were carried to the uterus by the blood, initially infecting the endometrium and subsequently spreading to the placenta and foetus [47]. *Brucella* were detected in phagosomes and the rough ER (RER) of chorioallantoic trophoblasts [6,22] and replicated within the RER causing hypertrophy of this organelle [6,22,34]. Infection of the chorionic trophoblast may happen through cell-to-cell lateral transfer from the infected erythrophagocytic trophoblasts [6]. Then, progression of infection may occur by new cell-to-cell transfer between trophoblasts or by trophoblastic necrosis and rupture, releasing brucellae to the extracellular spaces and further endocytosis by adjacent trophoblasts [6,48]. Macrophages and neutrophils are recruited to these ulcerated and necrotised areas [5]. There, macrophages become infected and brucellae replicate at high numbers within them and, together with neutrophils, induce an inflammatory reaction in the placenta [6,22,34]. Although the role of host immunity on the development of *Brucella*-induced placentitis has not been explored in the natural host, as has been performed for other reproductive pathogens such as *Chlamydia abortus* [49], a recent publication has reviewed the immune responses that are possibly involved in trophoblast necrosis and the derived gestational complications of brucellosis [50]. Trophoblast necrosis leads to chorioallantoic ulceration and the release of brucellae into the uterine lumen [6,34,47]. The proximity of placental capillaries to these ulcerated areas allows brucellae to access foetal circulation, spreading through the placentome structure and into the foetal tissues [34]. For all these reasons, trophoblasts have been suggested as the primary cell type involved in *Brucella* reproductive pathogenesis leading to abortion [6,34]. In the end, the resulting lesions, placentitis, and vasculitis, in both maternal and foetal tissues, may weaken the unions between foetal trophoblasts and the maternal epithelium, contributing to the death of the foetus and consequent abortion [47,51,52,53].

## 2. Placentation in *Brucella* Natural Hosts and Mouse

The study of *Brucella’s* systemic and reproductive pathogenesis in the natural host is greatly restricted due to their size, containment infrastructure, maintenance costs, and ethical aspects. Although mice are not natural hosts for relevant *Brucella* spp., this laboratory model is widely utilised in brucellosis research [54]. Replication profiles in the mouse spleen are valuable for determining variations in virulence among *Brucella* strains and live vaccines. The mouse model is also instrumental in assessing the potential protective efficacy of *Brucella* vaccine candidates. To a lesser extent, the pregnant mouse model (the focus of the present review) has been employed to study the abortifacient effect of *Brucella* species, mainly *B. abortus* and *B. melitensis*, with only one study on *B. suis*, one on *B. ovis,* and none on *B. canis*.

**Figure 2:**
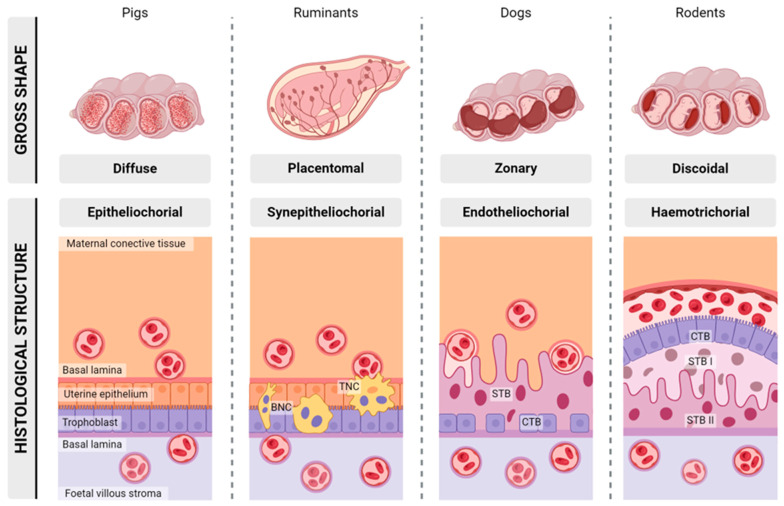
Comparative placentation based on gross shape and the histological interhaemal layers at the maternal–foetal interface of the placenta. BNC: Binucleated cell; TNC: Trinucleated cell; CTB: Cytotrophoblast; STB: Syncytiotrophoblast. (Created with Biorender.com).

To understand both the strengths and limitations of this murine model, it is crucial to comprehend the placental structures of the diversity of *Brucella* natural hosts and mice. The placenta is an organ that forms by the apposition of maternal and foetal (trophoblast; from the Greek *trephein*, to feed, and *blastos*, germinator) tissues whose function is to maximise the exchange of molecules (e.g., nutrients, debris, signalling factors, etc.) and to minimise immunological rejection by the maternal immune system while the foetus develops. To accomplish these key functions, a wide diversity of placentation types has arisen through mammalian evolution [55], which can be classified micro- and macroscopically according to their histological structures and shapes (Figure 2).

Suidae exhibit an epitheliochorial placentation characterised by the apposition of the foetal trophoblast (-*chorial*) to the maternal uterine epithelium (*epithelio*-), with a mean gestation period of 115 days [56,57]. In this placentation, there is minimal invasion of the uterine lining and no cell/tissue layers are removed (Figure 2). This way, the uterine epithelium comes into contact with the trophoblast and both layers interdigitate through microvilli, greatly increasing the exchange surface, which is further increased by the development of villi over the entire surface of the placenta in a diffuse pattern (Figure 2).

Ruminants (e.g., sheep, goats, and cows) exhibit a cotyledonary (with 20–150 placentome units) and synepitheliochorial placentation in which there is a fusion (*syn*-) of maternal epithelium (-*epithelio*-) and foetal trophoblasts (-*chorial*) (Figure 2) [56,57], with mean gestation periods of 147, 150, and 283 days for sheep, goats, and cows, respectively. Microscopically, the uterine epithelium (the caruncle) and the trophoblasts (the cotyledon) establish close contacts by the formation of interdigitations through microvilli that greatly increase the exchange surface. A population of mononuclear trophoblastic cells fuse together into binucleated cells that migrate and further fuse with the uterine caruncular epithelium forming foetomaternal trinucleated cells or syncytial plaques of bigger sizes (Figure 2), helping to transfer effector molecules towards the maternal side [58]. Macroscopically, this foetomaternal structure folds (further increasing exchange surface) into a delimitated structure, the placentome, the total number of which depends on the species.

Dogs (as do other carnivores) exhibit an endotheliochorial placentation in which trophoblastic cells (-*chorial*) form a syncytium that completely erodes the uterine epithelium, directly apposing to an originated amorphous interstitial layer around the persisting maternal endothelium (*endothelio*-) (Figure 2) [56,57]. This layer is not continuous in all its surface and some trophoblast–endometrium apposition can still be found in peripheral regions of the placenta. In this placentation type, the foetomaternal structure also develops villi and folding in order to increase the exchange surface and acquires a zonary distribution around the conceptus (Figure 2).

Rodents exhibit a discoidal labyrinthine haemotrichorial in which maternal blood (*haemo*-) bathes a three-cell-layered (-*tri*-) foetal trophoblast (-*chorial*) placentation (Figure 2) [56,57], with a mean gestational duration of 19–21 days. Microscopically, the uterine epithelium is completely eroded upon implantation and maternal blood directly bathes the foetal trophoblast formed by a syncytiotrophoblast population that establishes two cell layers surrounding the foetal capillaries (syncytiotrophoblast I and II) and a mononuclear trophoblast population (cytotrophoblasts) that lines the maternal blood sinuses (Figure 2) [59]. Macroscopically, this foetomaternal exchange structure folds into an amorphous network [60], called the labyrinth, that is structurally supported by the spongiotrophoblast and limits with the decidua (i.e., the maternal side of the placenta) through a lining of trophoblastic giant cells (TGC) known as parietal TGCs, a region known as the junctional zone. The decidua is another specific trait of haemochorial placentation; it is formed by the transformation of the endometrium prior to trophoblast invasion and during placenta formation and helps to anchor the placenta in order to channel maternal blood vessels into the placenta and to protect the developing embryo from external aggressions.

## 3. The Pregnant Mouse Model in *Brucella* Research

**Figure 3:**
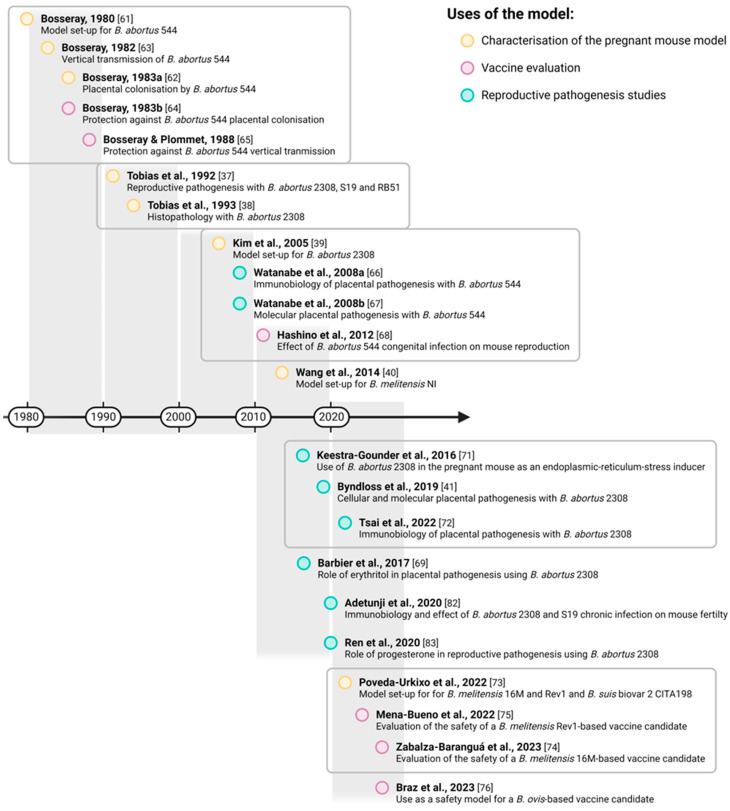
Chronology of the reviewed publications on brucellosis using the pregnant mouse model. References clustered within a box indicate studies performed by the same research group or related researchers [37,38,39,40,41,61,62,63,64,65,66,67,68,69,71,72,73,74,75,76,82,83]. (Created with Biorender.com).

The first steps into the evaluation of *Brucella*-induced reproductive pathogenesis in pregnant mice were carried out in the early 80s by Nicole Bosseray using *B. abortus* (Figure 3 and Table 1). These studies focused on characterising the *B. abortus* biovar 1 strain 544 infection profile in pregnant CD-1 mice as a tool to study *Brucella* reproductive pathogenesis [61,62], the processes involved in vertical transmission [63], and the role of vaccination in the prevention of *Brucella*-induced abortion [64,65]. Bosseray’s studies were followed by Tobias et al. (Figure 3), who characterised the pregnancy outcome of *B. abortus* biovar 1 strain 2308-infected mice from the histopathological point of view and then compared it with the outcome of the *B. abortus*-based S19 and RB51 vaccine strains [37,38].

Studies in the pregnant mouse model were not resumed until 2005 (Figure 3), when Kim et al. characterised the abortifacient effect of *B. abortus* 544 at different inoculation times during pregnancy and compared it with the effect of the S19 vaccine [39], in a similar way to that of Bosseray and Tobias et al. in the previous decades (Table 1). However, it appears that these studies were not considered by Kim et al. (“(sic)”: “no studies on the induction of abortion by Brucella have been conducted in the pregnant mouse”) [37,38,61,62,63,64,65]. This way, Kim et al.’s work was the main reference in the following decades for subsequent *Brucella* studies in the pregnant mouse model that focused on different aspects of *Brucella* reproductive pathogenesis during pregnancy [41,66,67,68,69,70,71,72]. Later (Figure 3), Wang et al. also characterised the dose-dependent abortifacient effect of a *B. melitensis* biovar 3 field strain (NI strain) (Table 1) [40].

Up until 2022 (Figure 3), few studies had assayed the abortifacient potential of live brucellosis vaccines, and the ones that did so focused only on *B. abortus*-based vaccines such as the S19 and/or RB51 (Table 1) [37,39,64]. In 2022, Poveda-Urkixo et al. characterised the abortion induction of several *Brucella* strains, the *B. melitensis* biovar 1 strain 16M and the Rev1 live attenuated vaccine strain, and the *B. suis* biovar 2 strain CITA198 (Table 1) [73]. Then, these authors used their model to evaluate the reproductive safety of two vaccine candidates on the *B. melitensis* 16M- and Rev1-backgrounds [74,75]. Coincidentally to these studies, the pregnant mouse was also used to study the abortifacient effect of the *B. ovis* strain ATCC^®^ 25840 as well as a *B. ovis*-based vaccine candidate (Table 1) [76].

This review of the literature indicates that the variety of publications that have investigated different aspects of *Brucella* reproductive pathogenesis in the pregnant mouse have employed a plethora of experimental designs: mouse strains, doses, routes of inoculation, infection at different times post-conception (p.c.), abortion/stillbirth readouts, etc., (Table 1). Thus, herein we compare the different experimental designs employed for the pregnant mouse model of reproductive brucellosis, focusing on *Brucella*-induced adverse gestational events and the use of the model for vaccine safety evaluation.

### 3.1. Methodological Aspects of the Model

#### 3.1.1. Mouse Pregnancy Outcome and Abortion Readout

In ruminants, abortions at mid–late gestation are the main clinical manifestation of *Brucella* infection in the field [77]. Abortions at this stage have been linked to hormonal and nutritional factors [31,78], but the underlying mechanisms are poorly understood, and it is possible that foetal deaths during the first trimester of pregnancy may go unnoticed [79]. This way, *Brucella*-induced abortions are mainly noticed at stillbirth [31], as happens with other abortifacient pathogens such as *C. abortus* or *Toxoplasma gondii* [80,81]. In the pregnant mouse model, the effects of *Brucella* infection may result in two different outcomes depending on the time of pregnancy at which abortion happens. On the one hand, early adverse pregnancy events may result in abortion and resorption of the still small conceptuses, resulting in a decreased litter size. On the other hand, late events may result in foetal death with no resorption, as the foetuses are mostly developed and of a considerable size, resulting in a decreased pup viability (with observable aborted foetuses/stillbirths).

In the literature, publications have typically expressed adverse pregnancy events in the pregnant mouse model only as a decrease in pup viability. Possible explanations for this may be that it was the only parameter investigated [41,67,71,72,82,83], that authors did not find a remarkable effect on litter sizes [39,40,66], or that reductions in litter sizes were unnoticed by the authors despite obtaining them in their results [73]. In the latter study, Poveda-Urkixo et al. noted that 2/5 pregnant mice inoculated with 10^6^ CFU/mouse of *B. melitensis* 16M at 4 days p.c. (Table 1) experienced complete abortions as foetal resorptions [73]. However, these two mice were not included in the determination of the litter size, which was therefore averaged over the remaining three mice, resulting in a litter size of 15.0 ± 3.6. This way, the calculation result is the highest litter size from all the *Brucella* strains studied, even higher than the PBS control (13.3 ± 0.6 pups/litter). If the calculation had included the two complete-abortion events, the litter size result would be 9.0 pups/litter, a value more coherent with the expected abortifacient effect of *B. melitensis* 16M. The phenomenon of litter size reduction was indeed noticed in a study by Hashino et al., who showed that *B. abortus* 544 congenitally infected mice (Table 1) exhibited a reduction in litter sizes from 14.92 to 7.65 pups on average [68].

**Table 1:** ^a^ Experimental conditions ^b^ and main findings of the reviewed studies on brucellosis pregnant mouse models.

Mouse Strain/ Mating Conditions	Time of Infection/ Time of Euthanasia	*Brucella* Strain, Administration Route and Dose (CFU/Mouse)	Main Findings
Bosseray, N. Colonization of Mouse Placentas by *Brucella abortus* Inoculated during Pregnancy. *Br. J. Exp. Pathol.* 1980, *61*, 361–368. [61]			
CD1 Mating: 1–2 in-house-born females were kept for 3 nights with 1 in-house-born male. Day 1 = Vaginal plug	Infection at 3, 7, 11, or 15 dpc Euthanasia at 18 dpc (15, 11, 7, or 3 dpi)	*B. abortus* 544 IP, 1.5 × 10^1–5^ IV, 2.5 × 10^1–4^ SC, 1.0 × 10^2–8^	- Placental colonisation was more intense within a 7–11 day p.c. temporal window. - No abortions or foetal death due to *Brucella* infection. - Placentae can be colonised independently of each other. - Colonisation was not dependent on the site of conceptus implantation within the uterus. - Pregnancy was not a more susceptible period for splenic infection. - The IP route of infection leads to a more efficient placental colonisation, followed by the IV and SC routes.
Bosseray, N. Mother to Young Transmission of *Brucella abortus* Infection in Mouse Model. *Ann. De Rech. Vet.* 1982, *13*, 341–349. [63]			
CD-1 Mating: as in Bosseray, 1980 [61]	Infection at 6 dpc Euthanasia at different times post-birth	*B. abortus* 544 Wb *B. abortus* 544 CO_2_-independent *B. abortus* 544 Strp-resistant IP, 1.5–1.8 × 10^5^	- 60% of pups were congenitally infected. - Infection acquisition through the consumption of milk was very scarce, as mammary gland colonisation was small. - The barrier effect of the placenta is individual of each one of them within the same dam, in line with Bosseray’s previous findings [47].
Bosseray, N. Kinetics of Placental Colonization of Mice Inoculated Intravenously with *Brucella abortus* at Day 15 of Pregnancy. *Br. J. Exp. Pathol.* 1983, *64*, 612–616. [62]			
CD-1, OF1 Mating: as in Bosseray, 1980 [61]	Infection at 15 dpc Euthanasia at 5, 10, 20, or 40-min or 1, 1.5, 2, 4, 6, 24, 48, or 72 h p.i.	*B. abortus* 544 IV, 1–2 × 10^4^	- Spleen colonisation happens earlier upon infection, but then splenic infection stabilises and the placenta rapidly become the main infection focus. - *Brucella* multiplication takes place at a higher rate in the placenta than in the spleen. - The generation time of *Brucella* at the placenta was estimated around 4–6 h, similar to that *in vitro*.
Bosseray, N. Vaccine and Serum-Mediated Protection against *Brucella* Infection of Mouse Placenta. *Br. J. Exp. Pathol.* 1983, *64*, 617–625. [64]			
CD-1, OF1 Mating: as in Bosseray, 1980 [61]	Infection at 7 or 15 dpc Euthanasia at 16 or 18 dpc	*B. abortus* 544 IP, 1.8–2.1 × 10^5^ IV, 1.8–4.5 × 10^4^/1.8–2.1 × 10^5^	- First publication to show that the induction of protection in pregnant mice had an impact on not only systemic but also placental infection. - Vaccination with peptidoglycan or lipopolysaccharide fractions killed *B. melitensis* H38 or living B19 vaccine protects pregnant mice against placental and splenic infection.
Bosseray, N.; Plommet, M. Serum- and Cell-Mediated Immune Protection of Mouse Placenta and Fetus against a *Brucella abortus* Challenge: Expression of Barrier Effect of Placenta. *Placenta* 1988, *9*, 65–79. doi:10.1016/0143-4004(88)90074-4. [65]			
OF1, DBA/2 Mating: as in Bosseray, 1980 [61]	Infection at 12 or 14 dpc Euthanasia at 3 or 5 dpi	*B. abortus* 544 IV, 1.8–4.5 × 10^4^/1.8–2.1 × 10^5^	- Foetuses were never colonised when the corresponding placentae were not. Foetal infection was dependent on the levels of placental infection.
Tobias, L.; Schurig, G.G.; Cordes, D.O. Comparative Behaviour of *Brucella abortus* Strains 19 and RB51 in the Pregnant Mouse. *Res. Vet. Sci.* 1992, *53*, 179–183. doi:10.1016/0034-5288(92)90107-D. [37]			
BALB/C	Infection at 9 dpc Euthanasia at 18 dpc (9 dpi)	*B. abortus* 2308 (IP, 10^5.7^) *B. abortus* S19 (IP, 10^7.5^) *B. abortus* RB51 (IP, 10^9.5^)	- A minimum dose of 10^7.5^ CFU/mouse of *B. abortus* S19 was needed to obtain splenic infection levels and produce lesions similar to those caused by 10^5.7^ CFU/mouse of *B. abortus* 2308. - *B. abortus* 2308, S19, and RB51 strains localised intracellularly within parietal TGCs.
Tobias, L.; Cordes, D.O.; Schurig, G.G. Placental Pathology of the Pregnant Mouse Inoculated with *Brucella abortus* Strain 2308. *Vet. Pathol.* 1993, *30*, 119–129. doi:10.1177/030098589303000204. [38]			
BALB/C Mating: in-house-born mice were individually mated. Day 1 = Vaginal plug	Infection at 9 dpc Euthanasia at 12, 14, 16, or 18 dpc (2, 5, 7, or 9 dpi)	*B. abortus* 2308 IP, 10^4.7/5.7/6/6.7^	- In-depth characterisation of histopathological progression of placental infection. - As infection progressed, brucellae replicated within trophoblasts, inducing a progressive necrosis of these cells and inducing the recruitment of macrophages and neutrophils, resulting in extensive placentitis. - Placental infection was restricted to the decidua and parietal TGCs with scarce involvement of the labyrinth region. - Within infected TGCs, brucellae were located in membrane-bound cisternae continuous with the RER and the perinuclear envelope. - In neutrophils, brucellae were typically found within phagosomes rather than RER cisternae.
Kim, S.; Dong, S.L.; Watanabe, K.; Furuoka, H.; Suzuki, H.; Watarai, M. Interferon-γ Promotes Abortion Due to *Brucella* Infection in Pregnant Mice. *BMC Microbiol.* 2005, *5*, 22. doi:10.1186/1471-2180-5-22. [39]			
ICR Mating: 6–10-week-old females individually mated to 6–10-week-old males. Day 0.5 = Vaginal plug	Infection at 3.5, 4.5, 6.5, 9.5, or 14.5 dpc Euthanasia at 18.5 dpc (15, 14, 12, 9, or 4 dpi)	*B. abortus* 544 *B. abortus* Δ*virB4* *B. abortus* S19 IP, 10^4^	- Highest foetal abortion (i.e., 98.4%) was obtained for inoculation of *B. abortus* 544 on day 4 p.c. Infection with *B. abortus* Δ*virB4* or S19 strains resulted in no abortions. - *B. abortus* 544-infection induces IFN-γ production, with a peak at 3 dpi, that in pregnant animals was associated with abortion induction. - IFN-γ production did not happen in Δ*virB4*- or S19-infected mice. - Brucellae were isolated from both aborted and alive foetuses or placentae at similar rates. - Brucellae were present within parietal TGCs, neutrophils, or free at the decidua.
Watanabe, K.; Iwai, N.; Tachibana, M.; Furuoka, H.; Suzuki, H.; Watarai, M. Regulated upon Activation Normal T-Cell Expressed and Secreted (RANTES) Contributes to Abortion Caused by *Brucella abortus* Infection in Pregnant Mice. *J. Vet. Med. Sci.* 2008, *70*, 681–686. doi:10.1292/jvms.70.681. [66]			
BALB/c, IFN-γ KO BALB/c Mating: as in Kim et al., 2005 [39]	Infection at 4.5 dpc Euthanasia at 18.5 dpc	*B. abortus* 544 *B. abortus* Δ*virB4* IP, 10^4^	- *B. abortus* 544 infection induces a steady increase of RANTES but not of MCP-1, which in pregnant animals was associated with abortion induction. - RANTES induction did not happen in the Δ*virB4*-infected mice.
Watanabe, K.; Tachibana, M.; Tanaka, S.; Furuoka, H.; Horiuchi, M.; Suzuki, H.; Watarai, M. Heat Shock Cognate Protein 70 Contributes to *Brucella* Invasion into Trophoblast Giant Cells That Cause Infectious Abortion. *BMC Microbiol.* 2008, *8*, 212. doi:10.1186/1471-2180-8-212. [67]			
ICR Mating: as in Kim et al., 2005 [39]	Infection at 4.5 dpc Euthanasia at 18.5 dpc	*B. abortus* 544 IP, 10^4^	- In vitro internalisation of *B. abortus* 544 within TGCs was dependent on Hsc70 present on the cell surface. - TGCs express IFN-γ receptors. - Anti-Hsc70 antibody treatment increases pup viability but does not affect splenic or placental bacterial burden.
Hashino, M.; Kim, S.; Tachibana, M.; Shimizu, T.; Watarai, M. Vertical Transmission of *Brucella abortus* Causes Sterility in Pregnant Mice. *J. Vet. Med. Sci.* 2012, *74*, 1075–1077. doi:10.1292/jvms.11-0566. [68]			
ICR Mating: as in Kim et al., 2005 [39]	Infection at 6.5 dpc Euthanasia at different times post-birth	*B. abortus* (strain not specified; presumably 544) IP, 10^4^	- Vertical transmission to the first-generation offspring was due to transplacental infection but not to contaminated milk consumption. - Congenital infection of first-generation offspring resulted in lower body weight and reduced fertility. - No vertical transmission was detected to the second-generation offspring, but they also had lower body weight and reduced fertility.
Wang, Z.; Wang, S.S.; Wang, G.L.; Wu, T.L.; Lv, Y.L.; Wu, Q.M. A Pregnant Mouse Model for the Vertical Transmission of *Brucella melitensis*. *Vet. J.* 2014, *200*, 116–121. doi:10.1016/j.tvjl.2013.12.021. [40]			
ICR Mating: as in Kim et al., 2005 [39]	Infection at 4.5 dpc Euthanasia at 18.5 dpc (14 dpi)	*B. melitensis* NI IP, 10^3–6^	- Bacterial recovery was higher as the dose increased. Bacterial burden was similar regardless of the alive/death status of the foetuses. - A minimum dose of 10^4^ CFU/mouse of *B. melitensis* NI was needed to observe gross placental lesions, but histological lesions were noted at every dose tested. - Placentae associated with aborted foetuses were pale, soft, and shrunken, while the ones associated with alive foetuses dark red, firm, and of expected size. - *B. melitensis* NI was found surrounding the nucleus within TGCs, which showed a small and vacuolated cytoplasm. - Heavy neutrophil infiltration with foci of necrosis that extended to the spongiotrophoblast.
Keestra-Gounder, A.M.; Byndloss, M.X.; Seyffert, N.; Young, B.M.; Chávez-Arroyo, A.; Tsai, A.Y.; Cevallos, S.A.; Winter, M.G.; Pham, O.H.; Tiffany, C.R.; et al. NOD1 and NOD2 Signalling Links ER Stress with Inflammation. *Nature* 2016, *532*, 394–397. doi:10.1038/nature17631. [71]			
C57BL/6 Mating: in-house mating. No further specifications	Infection at 5 dpc Euthanasia at 8, 12, and 18 dpc (3, 7, and 13 dpi)	*B. abortus* 2308 *B. abortus* 2308 ∆*vceC* IP, 10^5^	- *B. abortus* 2308-induced abortions and placentitis mediated, in part, by the ER-stress response through the NOD1/2-mediated unfolded protein response. - *B. abortus* 2308 ∆*vceC* (an effector molecule involved in the induction of ER stress within *Brucella*-infected cells) induces reduced abortion and placentitis levels without affecting placental bacterial burden.
Barbier, T.; Machelart, A.; Zúñiga-Ripa, A.; Plovier, H.; Hougardy, C.; Lobet, E.; Willemart, K.; Muraille, E.; De Bolle, X.; Van Schaftingen, E.; et al. Erythritol Availability in Bovine, Murine and Human Models Highlights a Potential Role for the Host Aldose Reductase during *Brucella* Infection. *Front. Microbiol.* 2017, *8*, 1088. doi:10.3389/fmicb.2017.01088. [69]			
C57BL/6 Mating: oestrus was synchronised 3 days before mating. Each female was mated with one male and then isolated. Adapted from Bosseray, 1982 and Kim et al., 2005 [39,63]	Infection at 6 and 14 dpc Euthanasia at 15 dpc (9 and 1 dpi)	*B. abortus* 2308 *B. abortus* 2308 ∆*eryA* *B. abortus* 2308 ∆*eryH* IP, 10^5^	- Only one of the erythritol-catabolism mutants (Δ*eryH*) was attenuated in foetuses, placentae, or foetal envelopes. - *B. abortus* 2308 was found almost exclusively within TGCs at the junctional zone.
Byndloss, M.X.; Tsai, A.Y.; Walker, G.T.; Miller, C.N.; Young, B.M.; English, B.C.; Seyffert, N.; Kerrinnes, T.; de Jong, M.F.; Atluri, V.L.; et al. *Brucella abortus* Infection of Placental Trophoblasts Triggers Endoplasmic Reticulum Stress-Mediated Cell Death and Fetal Loss via Type IV Secretion System-Dependent Activation of CHOP. *mBio* 2019, *10*, e01538–19. doi:10.1128/mBio.01538-19. [41]			
C57BL/6J Mating: as in Keestra-Gounder et al., 2016 [71]	Infection at 5 dpc Euthanasia at 18 dpc (13 dpi)	*B. abortus* 2308 *B. abortus* 2308 ∆*virB2* *B. abortus* 2308 ∆*vceC* IP, 10^5^	- *B. abortus* 2308 caused moderate–severe cell death of TGCs and infiltrating neutrophils in an ER-stress-dependent manner, while a Δ*vceC* or Δ*virB2* mutants induced an intermediate or zero cell-death phenotype, respectively. - VceC induces ER stress in TGCs through the activation of CHOP, triggering cell death. - ER-stress-mediated trophoblast death may not be the sole cause of abortion.
Adetunji, S.A.; Faustman, D.L.; Adams, L.G.; Garcia-Gonzalez, D.G.; Hensel, M.E.; Khalaf, O.H.; Arenas-Gamboa, A.M. *Brucella abortus* and Pregnancy in Mice: Impact of Chronic Infection on Fertility and the Role of Regulatory T Cells in Tissue Colonization. *Infect. Immun.* 2020, *88*, e00257-20. doi:10.1128/IAI.00257-20. [82]			
ICR Mating: oestrus was synchronised by caging of mice with male house bedding for 3–5 days	Infection 8 weeks prior to mating Euthanasia at 18 dpc	*B. abortus* S2308 *B. abortus* S19 *B. abortus* S2308 ∆*virB2* IP, 10^6^	- *B. abortus* 2308 chronically infected mice yield less successful pregnancies but with no effect on pup viability. - *Brucella* exhibit a tropism towards the uterus even in an absence of pregnancy. - Both *B. abortus* 2308 and S19, but not the ∆*virB2* mutant, are able to persistently colonise the uterus.
Ren, J.; Hou, H.; Zhao, W.; Wang, J.; Peng, Q. Administration of Exogenous Progesterone Protects Against *Brucella abortus* Infection–Induced Inflammation in Pregnant Mice. *J. Infect. Dis.* 2021, *224*, 532–543. doi:10.1093/infdis/jiaa722. [83]			
C57BL/6J Mating: as in Keestra-Gounder et al., 2016 [71]	Infection at 5 dpc Euthanasia at 18 dpc (13 dpi)	*B. abortus* 2308 IP, 10^5^	- *B. abortus* 2308 infection induces a decrease in progesterone synthesis by the placenta, resulting in higher intracellular survival, placentitis values (IFN-γ and IL-6 production), and abortion induction due to the inflammatory role of progesterone.
Tsai, A.Y.; Byndloss, M.X.; Seyffert, N.; Winter, M.G.; Young, B.M.; Tsolis, R.M. Tumor Necrosis Factor Alpha Contributes to Inflammatory Pathology in the Placenta during *Brucella abortus* Infection. *Infect. Immun.* 2022, *90*, e0001322. doi:10.1128/iai.00013-22. [72]			
C57BL/6J Mating: as in Kim et al., 2005 [39]	Infection at 5 dpc Euthanasia at 18 dpc (13 dpi)	*B. abortus* 2308 *B. abortus* 2308 ∆*virB2* *B. abortus* 2308 ∆*vceC* IP, 10^5^	- *B. abortus* 2308 induces the specific expression of a TNF-α (and IFN-γ) transcript in the placenta through the ER-stress response. - Anti-TNF-α antibody treatment partially restored foetal viability and resulted in reduced or absent placentitis and trophoblastic death without affecting bacterial recovery from the placenta.
Poveda-Urkixo, I.; Ramírez, G.A.; Grilló, M.-J. Kinetics of Placental Infection by Different Smooth *Brucella* Strains in Mice. *Pathogens* 2022, *11*, 279. doi:10.3390/pathogens11030279. [73]			
CD1 Mating: 5 females were mated with 1 male for 2 days	Infection at 4.5 ± 1 dpc Euthanasia at 5.5, 7.5, 9.5, 11.5, 14.5, and 18.5 ± 1 dpc (1, 3, 5, 7, 10, and 14 dpi)	*B. melitensis* 16M *B. melitensis* Rev1 *B. suis* biovar 2 CITA198 IP, 6–7 × 10^5^	- *B. melitensis* 16M and Rev1 replicated in similar numbers at the placentae, while *B. suis* biovar 2 replicated in lower numbers. - *B. melitensis* 16M and Rev1 showed high rates of vertical transmission, but macroscopic lesions were more clearly observed in *B. melitensis* 16M. *B. suis* biovar 2 resulted in minimal vertical transmission and macroscopic lesions. - *B. melitensis* 16M and Rev1 induced a Th1-like cytokine profile, while *B. suis* biovar 2 induced a minimal cytokine response. - *B. melitensis* 16M and Rev1 induced more severe and extensive placentitis than *B. suis* biovar 2.
Braz, H.M.B.; Silva, M.F.; Carvalho, T.P. de; Silva, L.A. da; Soares, J.B.; Costa, F.B.; Sossai, B.G.; Paixão, T.A. da; Santos, R.L. Pathogenesis of *Brucella ovis* in Pregnant Mice and Protection Induced by the Candidate Vaccine Strain *B. ovis* Δ*AbcBA*. *Vaccine* 2022, *40*, 4617–4624. doi:10.1016/j.vaccine.2022.06.044. [76]			
Balb/c	Infection at 5 dpc Euthanasia at 17 dpc (12 dpi)	*B. ovis* ATCC 25840 *B. ovis* Δ*abcBA* IP, 10^6^	- The physiological state during gestation favours uterine colonisation by *B. ovis*. - Immunisation with *B. ovis* Δ*abcBA* resulted in lower *B. ovis* wild-type recovery from the uterus and foetuses but not from the placentae. - No *B. ovis*-induced histopathological differences whether pregnant mice were vaccinated or not.
Mena-Bueno, S.; Poveda-Urkixo, I.; Irazoki, O.; Palacios, L.; Cava, F.; Zabalza-Baranguá, A.; Grilló, M.J. *Brucella melitensis* Wzm/Wzt System: Changes in the Bacterial Envelope Lead to Improved Rev1Δ*wzm* Vaccine Properties. *Front. Microbiol.* 2022, *13*, 908495. doi:10.3389/fmicb.2022.908495. [75]			
CD1 Mating: as in Poveda-Urkixo et al., 2022 [73]	Infection at 4.5 ± 1 dpc Euthanasia at 18.5 dpc (14 dpi)	*B. melitensis* Rev1 (IP, 10^6^) Rev1Δ*wzm* (IP, 10^7^)	- The Rev1Δ*wzm* vaccine candidate was not recovered from foetuses or placentae and induced lower placentitis than Rev1, with normal foetal viability. - Similar results regarding the safety of the vaccine in this murine model and the natural host (ovine).
Zabalza-Baranguá, A.; Poveda-Urkixo, I.; Mena-Bueno, S.; Ramírez, G.A.; De Bolle, X.; Grilló, M.J. Vaccine Properties of *Brucella melitensis* 16MΔ*wzm* and Reactivation of Placental Infection in Pregnant Sheep. *Vaccine* 2023, *41*, 1554–1566. doi:10.1016/j.vaccine.2023.01.017. [74]			
CD1 Mating: as in Poveda-Urkixo et al., 2022 [73]	Infection at 4.5 ± 1 dpc Euthanasia at 18.5 dpc (14 dpi)	*B. melitensis* Rev1 (IP, 10^6^) *B. melitensis* 16M (IP, 10^6^) *B. melitensis* 16MΔ*wzm* (IP, 10^7^)	- The *B. melitensis* 16MΔ*wzm* vaccine candidate was not recovered from foetuses or placentae and induced lower placentitis than *B. melitensis* 16M or Rev1, with normal foetal viability. - Contradictory results regarding the safety of the vaccine in this murine model and the natural host (ovine).

^a^ The colour code used in the heading of each reference corresponds to the different experimental uses of the pregnant mouse model in each publication, as in Figure 3 (yellow: characterisation of pregnant mouse model; purple: vaccine evaluation; blue: reproductive pathogenesis studies). ^b^ The experimental conditions were extracted from the materials and methods of the relevant publications. In cases where days post-infection or days post-conception were not provided, these were calculated from each other considering the average gestation in mouse. Abbreviations: CFU, colony forming units; dpc, days post-conception; dpi, days post-infection; (R)ER, (rough) endoplasmic reticulum; IP, intraperitoneal; IV, intravenous; TGC, trophoblastic giant cells; SC, subcutaneous; Strp, streptomycin.

#### 3.1.2. Inoculation Day

Regarding the inoculation time during pregnancy, although there is a current consensus on noting the morning after the overnight mating of the mice (at which the vaginal plug is noticed) as day 0.5 [84,85], there is some diversity in the enumeration of mouse gestation in the reviewed literature (beginning with Bosseray’s work in the 1980s), as well as in the mating protocols followed. Hence, in order to avoid misunderstandings in the comments and comparisons made in this review, the specific enumerations and mating conditions used in the literature are also summarised (when provided) in Table 1.

Bosseray initially screened several time points (3-, 7-, 11-, and 15-days p.c.) with a *B. abortus* 544 challenge in CD-1 pregnant mice (Table 1). After assessing the splenic and placental bacterial loads on day 18 p.c., she identified a temporal window from 7 to 11 days p.c. when the pregnant mouse placentae were more prone to colonisation by brucellae [61]. However, these different degrees of placental colonisation did not correlate with any abortion event, with only 4 perinatal deaths out of 330 pups born from infected dams [63]. Inoculation within Bosseray’s 7–11-days-p.c. temporal window was also executed in later studies by Tobias et al. [37,38]. In these papers, the raw abortion numbers resulting from intraperitoneal (IP) infection with *B. abortus* 2308 of 9-day-pregnant mice (Table 1) are difficult to interpret; however, the authors describe cases of autolysed or inviable foetuses. The ambiguous abortifacient effect exerted by *Brucella* in Tobias et al.’s studies may be related to the use of Balb/c mice that, as previously mentioned, yield lower litter sizes that could hinder the manifestation of adverse pregnancy outcomes. These mouse strains differ widely in their reproductive performance, with CD-1 mice yielding an average of 13.5 pups/litter and Balb/c mice yielding 5.8 pups/litter (JanvierLabs). The smaller litter sizes of Balb/c mice may limit the possibility of observing significant effects on the reproductive outcome of pregnant mice as models of *Brucella* reproductive pathogenesis, given the narrower range of adverse pregnancy events to be tracked. Nonetheless, Bosseray’s and Tobias et al.’s results are surprising, not only because they do not correlate with the abortion outcome of *B. abortus* in bovines but also because a later publication by Kim et al. showed that *B. abortus* 544 infection on day-9 p.c. (i.e., within Bosseray’s temporal window) did induce abortions in an ICR (equivalent to CD-1) pregnant mouse model [39]. In this study, several infection days (3, 4, 6, 9, and 14 days p.c.) were screened in ICR pregnant mice IP infected with 10^4^ CFU/mouse of *B. abortus* 544 (Table 1). While Bosseray found no abortion events regardless of the time of infection [61], Kim et al. found that a dose of 10^4^ CFU/mouse induced the abortion of a considerable proportion of foetuses (28.1–37.3%) when infecting at very early (i.e., day 3) or intermediate pregnancy time-points (i.e., days 6 and 9), an almost complete foetal abortion (98.4%) at the early 4 days p.c. and no abortion when infecting in late pregnancy (i.e., 14 days p.c.) [39]. In this regard, it is intriguing how such a high level of abortion could occur at 4 days p.c. but not at 3 or 6 days p.c. Furthermore, such an early adverse effect on mice pregnancy should result in early-stage abortions that, due to their reduced dimension, would be more prone to be reabsorbed resulting in a decreased litter size; however, this does not seem to be the case, as authors recorded 15.75 pups/litter in the group inoculated at 4 days p.c. that showed a 98% abortion rate.

Subsequent studies that, as previously mentioned, took Kim et al.’s findings [39] as reference, supported the results that *Brucella* induces adverse gestational events in pregnant mice infected on day-4 p.c. First, the same research group that conducted the Kim et al., 2005 study (Figure 3) showed that IP infection with *B. abortus* 544 at 10^4^ CFU/mouse on day-4 p.c. (Table 1) reduced the number of live foetuses from 12–13 to 2–3 on average^1^ [66,67]. Subsequently, another research group (Figure 3) showed that infection with *B. abortus* 2308 on day-5 p.c. (via IP at 10^4^ CFU/mouse; Table 1) reduced foetal viability from >90% in the control mice to 0–7.04%^2^ [41,71,72], findings also supported by a publication by Ren et al., who showed a reduction in pup viability to 2.91% when infecting mice on day-5 p.c. [83]. Similar to *B. abortus*, *B. melitensis* strains have also been described as reducing foetal viability, completely in the case of the 16M virulent strain, to a lesser extent (i.e., 35.4 ± 33.0%; nonetheless, this group exhibited a remarkable deviation of viability rates among individuals) for the Rev1 vaccine when inoculation (6–7 × 10^5^ CFU/mouse via IP; Table 1) is performed on day 4.5 ± 1 [73], or to an even lesser extent for a *B. melitensis* non-reference field strain at the same inoculation day [40].

To summarise, despite the 7–11 days p.c. window identified by Bosseray when the placentae were more prone to colonisation by *B. abortus* 544 (but not for the induction of abortions), earlier inoculation days (4–5 days p.c.) have been adopted in subsequent publications based on the results obtained by Kim et al. [39].

#### 3.1.3. Route of Administration

In most studies, the inoculation was performed through the IP route, except for Bosseray, who compared the suitability of the IP, intravenous (IV), and subcutaneous (SC) routes [61,62]. Bosseray evaluated the impact of the inoculation route on infection outcome and showed that the dose needed to infect 50% of the placentae increased from the IP (*ca.* 10^2^ CFU/mouse), to the IV (*ca*. 10^3^ CFU/mouse), to the SC routes (*ca*. 10^4.5^ CFU/mouse), the latter needing the highest dose [61]. Interestingly, the progression of infection in placenta and spleen resolved differently. The spleen was rapidly and heavily colonised in the early minutes after IV inoculation, as a reflection of the blood clearing function of this organ. However, the increase in bacterial burden eventually slowed down from the early hours p.i. onwards, indicating an active killing by phagocytes and the multiplication of the surviving bacteria. In contrast, although few brucellae localised in the placenta early in the infection, the organ rapidly became the main focus of bacterial replication, evidencing the remarkable tropism of *Brucella* for this organ [62]. This aspect of an asymmetrical placenta/spleen infection was also highlighted in later publications after IP inoculation of *B. abortus* 544 [39,66,67], *B. abortus* 2308 [37,38], *B. melitensis* NI [40], *B. melitensis* biovar 1 strain 16M and Rev1 [73,74,75], and *B. ovis* [76]. Interestingly, the higher degree of placental colonisation did not result in an equally high foetal infection [40,63], evidencing the invasion-limiting barrier function of the placenta [86].

Although the IP route has been the most widely used in *Brucella* mouse pregnant models, probably due to the ease of administration, further comparative research is needed to assess whether IP grants more reliable results in terms of reproductive effects than the SC, IV, or other routes of infection.

#### 3.1.4. Dose

The dose-dependent effect on placental infection was also evaluated in Bosseray’s pioneering studies [61]. It was demonstrated that increasing doses of *B. abortus* 544 resulted in increased levels of placental colonisation, whether through the IP, IV, or SC route of administration, ranging from 0.41 to 6.00 log (10^1^–10^5^ CFU/mouse), 0.81 to 5.02 log (10^1^–10^4^ CFU/mouse), and 0.99 to 5.92 log (10^2^–10^8^ CFU/mouse,^3^ respectively (Table 1). However, as previously mentioned for the inoculation day, these increasing levels of bacterial placental colonisation did not correlate with any abortion event [63].

The dose dependence of bacterial burden and abortion was later studied for *B. melitensis* in 2014 by Wang et al., who screened several infection doses (10^3^–10^6^ CFU/mouse) of *B. melitensis* biovar 3 strain NI (a field strain of bovine origin) in ICR pregnant mice infected at day-4 p.c. [40] (Table 1). Here, a dose-dependent effect was found not only on bacterial burden in spleens, placentae, and foetuses, as found by Bosseray [61], but also on the number of pregnant mice that experienced abortions/stillbirths. While a dose of 10^3^ CFU/mouse was insufficient to induce any abortion, doses ranges of 10^4^–10^5^ CFU/mouse caused stillbirth cases in 50% of pregnant mice and the dose of 10^6^ CFU/mouse caused stillbirths in the entirety of the mice population [40]. The highest dose of 10^6^ CFU/mouse was defined as the minimum dose needed to produce consistent severe placental lesions in pregnant mice, an aspect also described previously by Tobias et al. for *B. abortus* 2308 [38]. Yet, as already noted by Wang et al., comparing their abortion results with those of Kim et al.’s [39] reveals noticeable differences. Contrary to what would be expected for these two *Brucella* spp., while in Wang et al. the inoculation of 10^4^ CFU/mouse of *B. melitensis* NI resulted in 50% of the dams experiencing adverse reproductive events but only 0.04% of foetal death [40], the inoculation of the same dose of *B. abortus* 544 at the same stage of pregnancy by Kim et al. induced abortion events in 100% of the pregnant mice, with 98.4% of foetuses aborted [39]. In the face of this dichotomy, another publication from Kim et al.’s group using the same experimental design evidenced that inoculation of mice at 10^4^ CFU/mouse of *B. abortus* 544 (via IP on day 4 p.c.) resulted in 80.86% aborted foetuses^4^ [66]. Further studies using an increased dose of 10^5^ CFU/mouse (via IP on day 5 p.c.) also showed foetal abortion rates between 90 and 100%^5^ for *B. abortus* 2308 [41,71,72,83] or *B. melitensis* 16M [73].

Overall, doses of 10^4^–10^6^ CFU/mouse (depending on the infective species and/or strain) are needed to obtain significant rates of abortions in *Brucella*-infected mice. It has also been shown that vaccine strains require higher doses to cause comparable reproductive problems.

### 3.2. Reproductive Pathogenesis in the Pregnant Mouse

Tobias et al. carried out a comprehensive histopathological characterisation by infecting pregnant Balb/c mice with 10^6^ CFU/mouse of *B. abortus* 2308 via the IP route at day-9 p.c. and studying the pathology over the course of mouse pregnancy (12, 14, 16, and 18 days p.c.; i.e., 3, 5, 7, and 9 days p.i.) (Table 1) [38]. Macroscopically, placentae from infected mice showed a reduced weight from day 14 until the end of gestation. According to the authors, placentae associated with viable foetuses were dark red and firm (as in non-infected mice) but with a yellow rim of material at the periphery, while placentae supporting unviable (edematous or autolysed) foetuses were pale and shrunken. Microscopically, it was noted that *B. abortus* 2308 (as well as the S19 and RB51 vaccine strains), localised within TGCs at the placental decidua already at 3 days p.i. (i.e., 12 days p.c.) [37,38], as also happens within trophoblasts in the natural host [6,22]. This TGC targeting by *Brucella* in the mouse placenta has been widely described in later publications [39,40,41,66,73]. Within TGCs, *Brucella* localised and replicated surrounding the nucleus [40,73] inside membrane-bound cisternae with ribosomes that were continuous with normal RER and the perinuclear envelope [38]. The regions where brucellae were localised within TGCs presented no inflammatory infiltrate yet (i.e., at 3 days p.i.), although bacteria could be also found within neutrophils; within them, brucellae were likely present inside phagosomes rather than ER cisternae [38,39]. At 5 days p.i. (14 days p.c.), brucellae extensively covered the decidua basalis (i.e., proximal) and reached the spongiotrophoblast layer, where more infected TGCs could be found together with small foci of necrosis [38]. At 7 days p.i. (i.e., 16 days p.c.), the whole decidua exhibited massive bacterial colonisation in the extracellular environment, with occasional infected TGCs together with necrotic regions filled with phagocytising neutrophils [38]. Such massive bacterial growth in the decidua was also shown by fluorescence in term placentae of C57BL/6 pregnant mice IP infected with 10^5^ CFU/mouse *B. abortus* 2308 at day-6 p.c. [69]. At the innermost layers, the spongiotrophoblast also exhibited multiple foci of moderate–severe levels of necrosis with a lower degree of neutrophil infiltration [38]. At 9 days p.i. (i.e., 18 days p.c.), the extension of the pathology described for the previous time-point increased, involving all placental layers, with extensive neutrophil infiltration, a common finding in other publications [39,40,41,73,74,75], and lack of TGCs in severely affected placentae [38]. Brucellae were also found to cause infarction at different levels: inducing thrombosis of the uterine vessels [38], micro-thrombi, or larger infarcted areas of the labyrinth blood vessels [73]. Such infarction phenomena could account for the foci of coagulative necrosis described in the spongiotrophoblast [40,73]. Noticeably, the progressive lack of TGCs in severely affected areas of infected placentae found by Tobias et al. could be due to progressive apoptosis of this cell population as a result of brucellae replication [38], as noted in *B. melitensis* NI- [40] or *B. abortus* 2308-infected placentae [41]. This apoptosis of infected TGCs was shown to be mediated by the ER-stress-response cascade in a virulence-factor-dependent manner [41], resulting in the induction of TNFα in the placenta, which plays a key role in the development of placental pathology and abortion induction but not in the ability of bacteria to replicate within the placentae [72].

The dependence of *Brucella* reproductive pathogenesis on known virulence factors has been shown throughout the literature. One of *Brucella’s* main virulence factors is the VirB-T4SS (type 4 secretion system), which translocates bacterial effectors to the cytoplasm of host cells and is essential for *Brucella’s* intracellular survival, replication, and pathogenesis [25,87,88,89,90,91]. Due to the key role of *Brucella* T4SS, mutants in this system are widely used as an attenuation control in virulence studies. Kim et al. and Watanabe et al. noticed that inoculation with 10^4^ CFU/mouse of a *B. abortus* 544 Δ*virB4* mutant at day-4 p.c. (Table 1) resulted in no abortion phenomena, while the same dose of the wild-type strain resulted in >80% aborted foetuses, despite bacterial colonisation being similar between the two strain groups [39,66]. Accordingly, a study by Byndloss et al. showed ca. 8% and 97% aborted foetuses for *B. abortus* Δ*virB2* and the corresponding wild-type strain, respectively [41]. However, in contrast to the mentioned previous studies, a 2-log drop in bacterial placental recovery for the *virB2* mutant with regards to the wild-type strain was reported [41]. Byndloss et al. also demonstrated that the T4SS-dependent *Brucella* effector molecule VceC (VirB-coregulated effector C) is involved in the induction of the ER-stress cascade leading to the apoptosis of infected trophoblasts and abortion [41]. Infection with *B. abortus* 2308 Δ*vceC* (Table 1) resulted in a reduction in aborted foetuses (48.22–61.63% abortions^6^), but with no significant drop in bacterial recovery from the placenta as happened for the VirB mutant.

Likewise, the specific nutrient microenvironment found at the placenta, and thus the unique *Brucella* metabolism, has also been proposed as an important driver of *Brucella* reproductive organ tropism, placental replication, and abortion induction. Regarding this, a study by Barbier et al. showed that depletion of erythritol catabolism (a sugar alcohol proposed as a preferential carbon source for *Brucella* at the placenta) in *B. abortus* 2308 (administered at 10^5^ CFU/mouse via IP on day 6 and 14 p.c.; Table 1) resulted in reduced bacterial burdens in the placentae and foetuses, although no data on abortion induction were shown [69].

In the light of these studies, the reproductive pathogenic differences observed between virulent and attenuated strains open the possibility of using pregnant mice as a predictive model for the potential abortifacient effect of brucellosis vaccine candidates (Section 3.4).

### 3.3. Vertical Transmission

Bosseray found that in 60% of cases the pups of an infected dam were infected, and the infection persisted throughout the experiments (30 days) without progressing to cure or superinfection [63]. However, as pointed out by the author, infection clearance might have been expected, as it is observed both in the natural host [92,93] and in infected adult mice after at least 16 weeks [94]. Infected newborns showed no signs of illness compared to non-infected pups, with no differences in the weight gain during the first 30 days of life. However, a later publication by Hashino et al. (from the same research group as Kim et al.) contradicts Bosseray regarding the health consequences on the offspring of *Brucella* infection [63]. In this publication, the growth and reproductive ability of the offspring born from *B. abortus* 544-infected mice were investigated using a similar experimental design: IP infection of ICR mice with 10^4^ CFU/mouse at 6.5 days p.c. [68] (Table 1). In this study, mice born from infected mice exhibited a significantly lower weight gain over the 8 weeks of study and had reduced fertility. This congenitally infected generation gave birth to an also weakened and low-numbered second-generation offspring but did not transfer the infection, despite the first generation being actively infected at the moment of mating. Supporting these results, a more recent study by Adetunji et al. demonstrated that *B. abortus* 2308 (10^6^ CFU/mouse via IP; Table 1) 8-week chronically infected non-pregnant mice yielded less successful pregnancies (i.e., 30.18 ± 21.05% success vs. 83.51 ± 4.56% success for the PBS control^7^), despite not affecting the number of alive pups in the successful gestations [82]. Brucellosis transmission from infected dams to offspring, either in utero or perinatally, is a common event in the natural host. Moreover, latent infections acquired through vertical transmission, when infected animals appear healthy and remain seronegative (thus becoming a dangerous source of brucellosis transmission), have been reported in up to 10% of the offspring from *Brucella*-infected cattle or small ruminants [93,95,96,97].

### 3.4. Vaccine Safety Screening

The assessment of brucellosis live vaccine candidates requires costly and cumbersome assays in the natural host (i.e., ovine, bovine, or porcine models) in order to evaluate, among other things, the vaccine safety in pregnant animals. However, experiments in the natural host are restricted due to animal size, the need for special facilities, high costs, and ethical aspects, thus laboratory models are imperative for an initial screening of vaccine candidates, and different mouse models are routinely used for this purpose [54]. In this context, the use of the pregnant mouse model for the assessment of the abortifacient effect of the live attenuated *B. abortus* S19 or *B. melitensis* Rev1 vaccine strains is also present in the literature, although these studies are much scarcer than for other wild-type *Brucella* species and strains.

In the initial studies on the pregnant mouse model, Tobias et al. showed that a dose of 10^7.5^ CFU/mouse of the S19 vaccine strain (via the IP route on day-9 p.c.; Table 1) was needed to cause lesions or splenic and placental colonisations similar to those obtained with the inoculation of 10^5.7^ CFU/mouse of *B. abortus* 2308 [37]. Also, Kim et al. noted that the inoculation of pregnant mice with the S19 vaccine (via the IP route on day-4 p.c.; Table 1) reduced the foetal abortion rate to 0.01%, when *B. abortus* 544 induced a 98.4% of abortion in the same conditions, despite not finding remarkable differences in terms of bacterial placental colonisation [39].

Regarding Rev1, the infection kinetics of this vaccine were evaluated upon inoculation of 4 ± 1-day-pregnant mice (via IP with 6–7 × 10^5^ CFU/mouse; Table 1) in a recent publication by Poveda-Urkixo et al. [73]. Rev1 infection resulted in approximately 35% pup viability (although individual data ranged from 0 to 75%), while infection with *B. melitensis* 16M led to complete abortion, in line with the expected attenuation of the vaccine strain. Likewise, although a relevant mention was not detected in the article, Rev1 infection also resulted in a reduced litter size of 9 pups/litter when compared to the 13.3 pups/litter in the PBS controls. This litter size reduction was similar on average to that of the *B. melitensis* 16M strain but less aggressive in the sense that two mice from the *B. melitensis* 16M-infected mice experienced complete abortions with no pups present at term (see Section 3.1.1), while the events in the Rev1-infected mice, in the two worst cases, resulted in litter sizes of four or nine pups [73]. The results in this study also evidence the differential progression of bacterial colonisation of the spleen and placentae originally mentioned by Bosseray, with the spleen being heavily colonised at early times p.i. and then decreasing to stable values and the placentae being colonised at lower levels but experiencing a steady increase in bacterial loads [62]. At term (i.e., 14 days p.i. or 18 days p.c.), the Rev1 bacterial burden in the placentae was in line with previous findings for other *Brucella* spp. and strains, with values between 6.43 and 8.44 log (CFU/g) [73,74,75]. The characterisation of both the *B. melitensis* Rev1 and 16M strains allowed the authors to further evaluate a vaccine candidate with a deleted *wzm*, which is involved in the synthesis of *Brucella* lipopolysaccharide [74,75]. In both backgrounds, the *wzm* mutant showed similar splenic colonisation values (slightly reduced for the *wzm* candidates) than the respective parental strains but, surprisingly, a complete absence of placental colonisation; the gestational outcome of the infected pregnant mice in terms of pup viability or litter size were not evidenced in this study. In these two publications, the behaviour of the corresponding vaccine candidates was further assessed in the ovine natural host, whereas the Rev1Δ*wzm* candidate showed similar safety results in terms of absence of abortifacient effect and bacterial recovery from infected dams [75], the 16MΔ*wzm* candidate was recovered from delivered placentae from infected ewes as opposed to the previous findings in the pregnant mouse model in this publication [74].

Finally, another publication evaluated the safety and abortion protection of a *B. ovis* ATCC^®^ 25840 mutant of an ABC transporter required for the intracellular survival and in vivo pathogenesis of *B. ovis* in mice and the natural host [76]. Although this study did not assess the safety of the candidate itself in pregnant animals, as no placental colonisation or abortion induction were tracked or demonstrated, it did show that the candidate induced reduced histopathological lesions in the placentae and uteruses of pregnant mice.

## 4. Concluding Remarks

In the light of this review, it is important to remark on the discrepancies regarding the presence/absence of abortions or litter reductions even for very similar experimental designs and even with the same *Brucella* spp. and/or strains. In addition, emphasis should be placed on the use of homogeneous mating conditions and the reporting of adverse pregnancy outcomes for *Brucella* infection, not only in terms of reduced pup viability but also including the effect on litter size or other informative gestational indicators. Nonetheless, there is sufficient evidence in the literature to support the notion that the pregnant mouse model is useful to study *Brucella*-induced placentitis and abortions, as shown by the histopathological progression of placental infection and the involvement of known *Brucella* virulence factors (e.g., VirB-T4SS or VceC).

The review on the experimental conditions employed through the literature shows that the IP inoculation of large-litter-size mice (such as ICR/CD-1) in the first half of gestation with *B. abortus* or *B. melitensis* induces abortion in pregnant mice, allowing the study of their reproductive pathogenesis. Regarding the dose, while 10^4^–10^5^ CFU/mouse has been established as the optimal dose for *B. abortus* biovar 1, the scarcity of publications, together with the use of different strains (from different biovars), makes it difficult to draw conclusions for *B. melitensis* and suggests that a dose close to 10^6^ CFU/mouse may be necessary to achieve foetal abortion rates comparable to those of *B. abortus*. Therefore, work with the pregnant mouse model may need to be standardised on a species- and strain-specific basis.

Concerning the establishment of the pregnant mouse model as a preliminary screening for vaccine safety improvement, the different protocols employed, together with the conflicting results obtained in mice and ewes, question the utility of the current mouse model and call for its optimisation. In this context, the study of brucellosis vaccines may greatly benefit from the retrospective or parallel evaluation of new vaccine candidates both in the mouse and the natural host (e.g., ovine), as recently performed by Mena-Bueno et al. and Zabalza-Baranguá et al. [74,75]. Nevertheless, as noted by these authors, the pregnant mouse model failed to replicate the reproductive safety outcome observed for at least one of the vaccine candidates in the ovine natural host [74], leading to the conclusion that work is still needed for a further refinement of the model as a tool for vaccine safety screening.

Finally, considering the diverse placentation types of the different *Brucella* spp. hosts, the standardisation of the pregnant mouse model for species other than *B. abortus* and *B. melitensis*, such as zoonotic *B. suis* or *B. canis*, will broaden the understanding of the pathogenicity of *Brucellaceae*. This may also contribute to the development of reproductively safe vaccines for different *Brucella* hosts.

## Data Availability

No new data were created or analysed in this study. Data sharing is not applicable to this article.

## Notes

1 When not provided, data were extracted from graphs using the informatic tool PlotDigitizer, 3.1.5, 2024, https://plotdigitizer.com, accessed on 27 February 2024.

2 See note 1 above.

3 See note 1 above.

4 See note 1 above.

5 See note 1 above.

6 See note 1 above.

7 See note 1 above.
